# Global Disruption of α2A Adrenoceptor Barely Affects Bone Tissue but Minimizes the Detrimental Effects of Thyrotoxicosis on Cortical Bone

**DOI:** 10.3389/fendo.2018.00486

**Published:** 2018-08-28

**Authors:** Gisele M. Martins, Marília B. C. G. Teixeira, Marcos V. Silva, Bianca Neofiti-Papi, Manuela Miranda-Rodrigues, Patricia C. Brum, Cecilia H. A. Gouveia

**Affiliations:** ^1^Department of Anatomy, Institute of Biomedical Sciences, University of São Paulo, São Paulo, Brazil; ^2^Department of Morphology, Federal University of Espírito Santo, Vitória, Brazil; ^3^Department of Morphology, Federal University of Sergipe, Aracaju, Brazil; ^4^School of Medicine, University of São Paulo, São Paulo, Brazil; ^5^University of Western Ontario, London, ON, Canada; ^6^School of Physical Education and Sport, University of São Paulo, São Paulo, Brazil

**Keywords:** thyroid hormone, thyrotoxicosis, sympathetic nervous system, α2A-adrenoceptor, cortical bone, trabecular bone, bone remodeling

## Abstract

Evidence shows that sympathetic nervous system (SNS) activation inhibits bone formation and activates bone resorption leading to bone loss. Because thyroid hormone (TH) interacts with the SNS to control several physiological processes, we raised the hypothesis that this interaction also controls bone remodeling. We have previously shown that mice with double-gene inactivation of α2A- and -adrenoceptors (α2A/2C-AR^−/−^) present high bone mass (HBM) phenotype and resistance to thyrotoxicosis-induced osteopenia, which supports a TH-SNS interaction to control bone mass and suggests that it involves α2-AR signaling. Accordingly, we detected expression of α2A-AR, α2B-AR and α2C-AR in the skeleton, and that triiodothyronine (T3) modulates α2C-AR mRNA expression in the bone. Later, we found that mice with single-gene inactivation of α2C-AR (α2C-AR^−/−^) present low bone mass in the femur and HBM in the vertebra, but that both skeletal sites are resistant to TH-induce osteopenia, showing that the SNS actions occur in a skeletal site-dependent manner, and that thyrotoxicosis depends on α2C-AR signaling to promote bone loss. To further dissect the specific roles of α2-AR subtypes, in this study, we evaluated the skeletal phenotype of mice with single-gene inactivation of α2A-AR (α2A-AR^−/−^), and the effect of daily treatment with a supraphysiological dose of T3, for 4 or 12 weeks, on bone microarchitecture and bone resistance to fracture. Micro-computed tomographic (μCT) analysis revealed normal trabecular and cortical bone structure in the femur and vertebra of euthyroid α_2A_-AR^−/−^ mice. Thyrotoxicosis was more detrimental to femoral trabecular bone in α2A-AR^−/−^ than in WT mice, whereas this bone compartment had been previously shown to present resistance to thyrotoxicosis in α2C-AR^−/−^ mice. Altogether these findings reveal that TH excess depends on α2C-AR signaling to negatively affect femoral trabecular bone. In contrast, thyrotoxicosis was more deleterious to femoral and vertebral cortical bone in WT than in α2A-AR^−/−^ mice, suggesting that α2A-AR signaling contributes to TH actions on cortical bone. These findings further support a TH-SNS interaction to control bone physiology, and suggest that α2A-AR and α2C-AR signaling pathways have key roles in the mechanisms through which thyrotoxicosis promotes its detrimental effects on bone remodeling, structure and resistance to fracture.

## Introduction

Thyroid hormone (TH) is recognized as an important regulator of bone remodeling, having a key role in the maintenance of bone mass and bone integrity (1). TH affects the skeleton indirectly, modulating the expression or action of other hormones and factors, such as GH and IGF-1 (2, 3), but also acts directly in the skeleton, controlling the proliferation, differentiation and/or activity of the main skeletal cells (4–7). Thyroid hormone receptors (TR) were detected in osteoblasts (8–11), osteocytes (9), osteoclasts (9, 12) and chondrocytes (13). In conditions of TH deficiency, both bone formation and resorption are decreased, leading to a state of low bone turnover. In this condition bone mass may be slightly increased or unchanged (14–16). In contrast, TH excess increases both osteoblastic and osteoclastic activities, but the latter is favored, leading to negative balance of calcium and bone loss (17–20). Therefore, thyrotoxicosis is an established cause of secondary osteoporosis, due to high bone turnover, with accelerated bone loss (16, 21, 22).

Over the last two decades, data has uncovered the sympathetic nervous system (SNS) as another potent regulator of bone remodeling (23). Evidence shows that SNS activation increases bone resorption and decreases bone formation leading to bone loss (24, 25), in a process that involves β2 adrenoceptors (β2-AR), expressed in osteoblasts (26, 27). However, studies by our group showed that female mice with global double inactivation of α2A and α2C adrenoceptors (α2A/C-AR^−/−^ mice) present a striking high bone mass (HBM) phenotype along with improved resistance to fracture, increased bone formation rate and decreased bone resorption, regardless of presenting increased sympathetic outflow and, therefore, a chronic increase in serum levels of catecholamines (28). The α2-adrenoceptor (α2-AR) family comprises α2A-, α2B- and α2C-AR subtypes (29–31), which are expressed in presynaptic membranes of adrenergic neurons, where they act as autoreceptors (receptors stimulated by the neurotransmitter released by the neuron where they are located) inhibiting the release of catecholamines (32, 33), especially norepinephrine (NE). Therefore, α2A/C-AR^−/−^ mice represent a mouse model of chronic sympathetic hyperactivity (34). Thus, the HBM phenotype in these animals revealed that β2-AR is not the only adrenoceptor involved in the control of bone turnover and raised the hypothesis that α2-AR subtypes also mediate SNS signaling in the skeleton (28). Accordingly, we and others found expression of α2A-, α2B- and α2C-AR in osteoblasts (28, 35, 36), osteoclasts, osteocytes and chondrocytes (28). In addition, *in vitro* findings reveal direct α2-AR signaling-mediated actions of the SNS in the skeleton (28, 37).

An important characteristic of TH is its interaction with the SNS to regulate several physiological processes (38). It is well known that TH-SNS interactions are necessary for maximum thermogenesis, lipolysis and lipogenesis (39). Interestingly, several clinical manifestations of thyrotoxicosis are indicative of increased adrenergic activity, such as tachycardia, increased cardiac output, increased glycogen and lipid mobilization, enhanced thermogenesis, tremor, hyperkinetic behavior, and sweating (40). In contrast, responses to adrenergic stimulation are low or blunted during hypothyroidism, leading, for example, to cold intolerance and limited metabolic responses to exercise (38, 41). Considering that both SNS hyperactivity and thyrotoxicosis have osteopenic effects, we raised the hypothesis that a TH-SNS interaction also occurs to regulate bone remodeling. The fact that treatment of hyperthyroid patients with propranolol, a β adrenoceptor antagonist, corrects the thyrotoxicosis-induced hypercalcemia (42) and decreases the urinary excretion of hydroxyproline, a biochemical marker of bone resorption (43), support a possible TH-SNS interaction to control bone remodeling, in addition to suggest that this interaction depends on the β-AR signaling pathway.

Recent studies by our group further support the hypothesis of a TH-SNS interaction to control bone remodeling, and reveals that α2-AR signaling has an important role in this interaction (28, 37, 44). We found that young adult female α2A/2C-AR^−/−^ mice are resistant to the deleterious effects of thyrotoxicosis on bone mass, on cortical and trabecular bone microarchitecture, and on bone resistance to fracture (37). In addition, *in vitro* studies bring evidence that TH-SNS interactions are likely to occur locally in the skeleton, via α2A-AR and/or α2C-AR signaling. Later, with the aim of discriminating the roles of the different α2-AR isoforms, we evaluated the bone phenotype of mice with the single gene inactivation of α2C-AR subtype (α2C-AR^−/−^) (44), which mRNA expression had been previously shown to be downregulated by TH in the femur (37). While α2A/2C-AR^−/−^ mice present a generalized phenotype of HBM (28), we found that α2C-AR^−/−^ animals present higher trabecular bone mass in the vertebra and lower trabecular bone mass in the femur (when compared with WT mice), which was accompanied by decreased bone strength in the femur and tibia (44). This heterogeneous bone phenotype of α2CAR^−/−^ mice reinforces the hypothesis that the SNS regulates bone remodeling and structure, via α2C-AR signaling, and suggests that this regulation is not the same across the skeleton, but rather may vary depending on the skeletal site. In spite of this heterogeneous bone phenotype, α2C-AR^−/−^ mice present resistance to the thyrotoxicosis-induced bone deterioration in both skeletal sites (femur and vertebra), likewise α2A/2C-AR^−/−^ mice (37, 44). These findings strongly suggest that the mechanism of action of TH to promote bone loss depends on α2C-AR subtype signaling. To confirm this hypothesis, in the present study, we characterized the bone phenotype of mice with global gene inactivation of only α2A-AR subtype, and the skeletal responses of these single KO animals (α2A-AR^−/−^ mice) to a 4- or 12-week-long-chronic condition of thyrotoxicosis.

## Materials and methods

### Animals and treatment

All the experimental procedures were carried out in accordance with the ethical principles and guidelines for animal research set by the Brazilian Society of Animal Experimentation, and were approved by the Ethics Committee on Animal Use (ECAU) of the Institute of Biomedical Sciences, University of São Paulo (protocol number 35/page 85/book 02). A cohort of female congenic C57BL/6J (B6) mice with gene inactivation of α2A-adrenoceptors (α2A-AR^−/−^) (34) and their wild-type (WT) controls (B6) were studied. All animals were 30 days old at baseline. The animals were kept under light- and temperature-controlled conditions (alternating cycles of light/dark for 12 h at a temperature of approximately 25°C), with *ad libitum* access to food and water. Thyrotoxicosis was induced by daily and i.p. administration of T3 (Sigma, St Louis MO, USA), at a dose of 7 μg T3/100g•body weight (BW)/day, which is equivalent to 20 times the physiological dose of T3 per day. T3-untreated (-) animals received daily i.p. injections of saline. Animals were treated with T3 or saline for 4 or 12 weeks, at the same time of the day each day. Animals were weighed once a week to follow changes in BW over the experimental period, in order to indirectly monitor a thyrotoxic state and with the purpose of adjusting the amount of T3 to be administered, to maintain the supraphysiological dose (20xT3), during the whole treatment period. Female mice were grouped (7 animals per group) as follows: WT and α2A-AR^−/−^ (T3-untreated animals, receiving saline), WT+T3 and α2A-AR^−/−^ + T3. At the end of the treatment period, animals were euthanized by exposure to CO2.

### Serum T3 and T4 assay

In order to confirm a thyrotoxic state, serum levels of T3 and thyroxine (T4) were measured at the end of the experimental period. Just after euthanasia, blood was collected by cardiac puncture. Serum was isolated by centrifugation and serum levels of total T4 and T3 were measured using radioimmunoassay commercial kits (RIA-gnost T4 and RIA-gnost T3; CIS Bio International, Gif-sur-Yvette, France). For the T4 and T3 assays, standard curves were built in our laboratory with a pool of charcoal-stripped mouse serum. Blood samples were always collected 2 h after the last T3 administration.

### Fat, skeletal muscles and heart mass

Considering the characteristic effects of thyrotoxicosis on fat mass, skeletal muscle mass and heart mass (45, 46), we measured these parameters at the end of the treatment period to also confirm a thyrotoxic state. Immediately after euthanasia, the axillar and retroperitoneal fat pads; the extensor digitorum longus (EDL), gastrocnemius and rectus femoris muscles; and the heart were dissected out and weighed for wet-mass determination. The skeletal muscles and heart samples were dehydrated at 60°C for 48 h and weighed again for dry-mass determination. Fat and heart masses were expressed in milligrams per gram of BW, whereas muscle masses were expressed inn milligrams per tibial or femoral length.

### Micro-computed (μCT) analysis of the femur and L5

Bone structural parameters of the right femur and the fifth lumbar vertebra (L5) were obtained using the μCT unit Skyscan 1174 (Bruker MicroCT, Kontich, Belgium), and the CtAn Software, version 1.5 (Bruker MicroCT). The X-ray settings were standardized to 100 kV for the baseline specimens, with an exposure time of 590 ms. A 0.05-mm-thick aluminum filter and a beam-hardening algorithm were used to minimize beam-hardening artifacts. The vertebral body of L5 and the distal metaphysis of the femur were the selected regions of interest (ROI). The trabecular bone parameters analyzed are trabecular volume (BV/TV); trabecular number (Tb.N); trabecular thickness (Tb.Th); trabecular separation (Tb.Sp); structure model index (SMI), which indicates the prevalence of rod-like (cylinder) and plate-like trabeculae (an ideal plate and cylinder have SMI values of 0 and 3, respectively) (47); trabecular pattern factor (Tb.Pf), a trabecular connectivity index (lower indexes indicate better connectivity of the trabecular structure) (48); trabecular porosity (Tb. Po); and trabecular bone mineral density (BMD). The cortical bone parameters analyzed are the total tissue area (T.Ar), which is the total area delimited by the periosteum, including the medullary and cortical area; cortical bone area (Ct.Ar); medullary area (Ma.Ar), cortical bone thickness (Ct.Th); periosteal perimeter (Ps.Pm); endocortical or endosteal perimeter (Ec.Pm); cortical porosity (Ct.Po), and cortical BMD.

### Three-point bending test of the femur

The right femurs were dissected and submitted to the three-point bending test, using the Instron testing system Model 3344 (Instron Corporation, MA, USA). During the test, the anterior cortex of the femur was placed in compression and the posterior cortex in tension. The distal and proximal extremities of the femur were supported on two anvils, spaced by a distance that equals the half of the femoral length (femoral length/2). A force was applied perpendicularly to the longitudinal axis of the bone, at the midpoint between the two supports, in the anteroposterior direction of the femur, by a crosshead, at a constant velocity of 5 mm/min, until the bone completely ruptured. To stabilize the specimen, a small preload (5% of the average maximum load) was applied before real testing. The applied force and the displacement of the crosshead were monitored and registered, at a sampling rate of 80 Hz, by the Bluehill software (version 3, Instron Corporation). The following biomechanical parameters were obtained and evaluated: Maximum load (N), which corresponds to the highest force applied during the test (maximum force at which the bone is able to withstand); tenacity (mJ), which is the bone's ability to withstand a fracture; and stiffness (N/mm), a measure of the rigidity of the bone tissue.

### Real-time PCR

Expression of α2A-AR, α2B-AR, α2C-AR, β1-AR, and β2-AR were determined by real-time PCR in the whole femur. The femurs were dissected and then crushed in a steel mortar and pestle set (Fisher Scientific International, Inc., Hampton, NH) precooled in dry ice. The crushed bones were transferred to microfuge tubes precooled in ice and total RNA was extracted using Trizol (Invitrogen, Carlsbad, CA), following the manufacturer's instructions. Total RNA was reverse transcribed using RevertAid-H-Minus M-MuLV Reverse Transcriptase (Fermentas, Hanover, MD) to synthesize the first-strand cDNA, which was used as a template. SYBR Green Super Mix (Applied Biosystems, Warrington, UK) was used for the real-time PCR using the ABI Prism 7500 sequence detector (Applied Biosystems, Foster City, CA). All primers used in this study [α2A-AR, forward (F): CGC AGG CCA TCG AGT ACA A and reverse (R): GAT GAC CCA CAC GGT GAC AA (NM_007417.3); α2B-AR, forward (F): TCC CTC TGG GAG GCA AGT G and reverse (R): GGC CAG GAT TCC AGA CCA TT (NM_ 009633.3); α2C-AR, F: CAT GGG CGT GTT CGT ACT GT and R: CAG GCC TCA CGG CAG ATG (NM_007418.3); β1-AR, forward (F): TCG TCC GTC GTC TCC TTC TAC and reverse (R): ACA CCC GCA GGT ACA CGA A (NM_007419); β2-AR, forward (F): GCC ACG ACA TCA CTC AGG AAC and reverse (R): CGA TAA CCG CAC TGA GGA TGG (NM_007420); 18S, F: GTA ACC CGT TGA ACC CCA TT and R: CCA TCC AAT CGG TAG TAG CG (NM_11188)] were designed using the Primer Express software (Applied Biosystems^TM^) and were synthesized (Integrated DNA Technologies, Coralville, IA) specifically for real-time PCR. All CT values were normalized using 18S as the internal control, which was validated for this study, showing to be stable (its expression did not vary due to mice lineage or T3 treatment). Relative gene expression quantification was assessed by the CT method, as described previously by Livak and Schmittgen (49). The final values for samples are reported as fold induction relative to the expression of the control, with the mean control value being arbitrarily set to 1.

### Statistical analysis

The statistical significance was determined by the Two-way analysis of variance (ANOVA), followed by the Tukey's test. Results were expressed as mean ± standard error of the mean (SEM). For all tests, *p* < 0.05 was considered statistically significant. For the statistical tests, we used the GraphPad Instat Software (GraphPad Software, Inc., San Diego, CA, USA).

## Results

### Effect of T3 treatment on serum levels of T3 and T4

**Figure 1:**
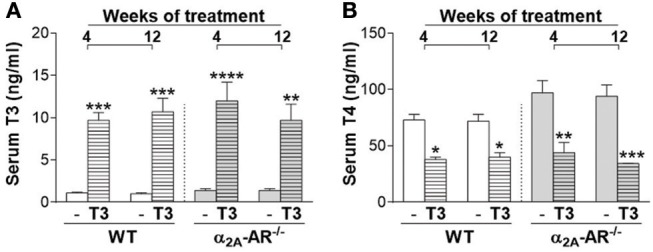
Serum levels of T3 and T4 in α_2A_-AR^−/−^ and WT mice. Animals were treated with a supraphysiological dose of T3 (7 μg/100 g BW/day) or saline (-) for 4 or 12 weeks, by daily i.p. injections. Values are expressed as mean ± SEM (*n* = 7 per group). ^*^P < 0.05, ^**^p < 0.01, ^***^p < 0.001, and ^****^p < 0.0001 vs. the respective saline-treated mice, by Two-way ANOVA followed by Tukey's test.

Serum levels of T3 were significantly higher (7- to 11-fold) in WT and α2A-AR^−/−^ animals treated with T3 for 4 or 12 weeks, when compared with their respective saline-treated controls (Figure 1A). On the other hand, T4 concentrations (Figure 1B) were significantly lower (44–64%) in WT and α2A-AR^−/−^ animals treated with T3 (vs. saline-treated animals), which reflects the suppression of the hypothalamic-pituitary-thyroid (HPT) axis promoted by T3 excess (50), and confirms a thyrotoxic state in both WT and KO animals. Serum levels of T3 and T4 were not different between WT and α2A-AR^−/−^ animals.

### Effect of T3 treatment on body composition and heart mass

**Figure 2:**
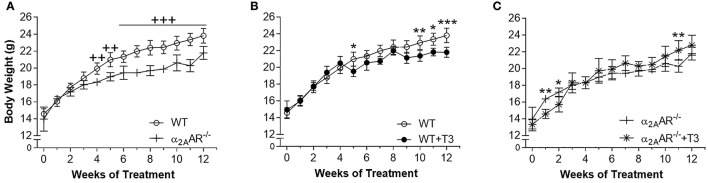
Effect of thyrotoxicosis on body weight of α_2A_-AR^−/−^ and WT mice. Animals were treated with a supraphysiological dose of T3 (7 μg/100 g BW/day) or saline for 12 weeks, by daily i.p. injections. Body weight was measured every week. Values are expressed as mean ± SEM (*n* = 7 per group). ^++^*P* < 0.01, and ^+++^*p* < 0.001 vs. WT; and ^*^p < 0.05, ^**^p < 0.01, and ^***^p < 0.001 vs. the respective saline-treated mice, by Two-way ANOVA followed by Tukey's test.

**Figure 3:**
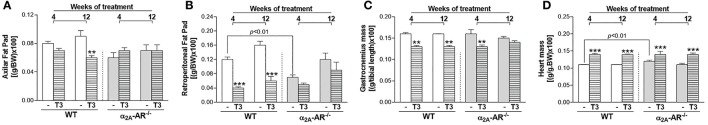
Effect of thyrotoxicosis on fat mass, muscle mass and heart mass of α_2A_-AR^−/−^ and WT mice. Animals were treated with a supraphysiological dose of T3 (7 μg/100 g body mass/day) or saline (-) for 4 or 12 weeks, by daily i.p. injections. Values are expressed as mean ± SEM (*n* = 7 per group). ^**^P < 0.01 and ^***^p < 0.001 vs. the respective saline-treated mice, by Two-way ANOVA followed by Tukey's test. *P*-values above the bars indicate differences between WT and KO mice.

To indirectly confirm a thyrotoxic state, we evaluated the effect of T3 treatment on body composition and heart mass. Figure 2A shows that α2A-AR^−/−^ mice presented 8–13% lower BW than WT mice from weeks 4 to 12 of this study, when animals were in the age range of 58–114 days. T3 treatment lead to lower BW in WT animals (Figure 2B), on weeks 5, 10, 11, and 12 of treatment (7–8%). In α2A-AR^−/−^ animals (Figure 2C), BW decreased 11 and 9% after 1 and 2 weeks of T3 treatment, respectively; but then tended to be higher in T3 treated KO animals as compared with saline-treated KO mice. In fact, on week 11, BW was 9% higher in KO mice treated with T3. In WT animals, T3 treatment significantly decreased the axillar fat mass, after 12 weeks of treatment (33%); and the retroperitoneal fat mass, after 4 and 12 weeks of treatment (66 and 62%, respectively), which was not observed in KO animals (Figures 3A,B). The retroperitoneal fat mass showed to be 42% lower in euthyroid KO animals vs. euthyroid WT animals by the end of the 4th week of this study, when animals were 58 days old (Figure 3B), but later these differences were not present anymore. Four weeks of treatment with T3 did not significantly affect the mass of EDL and rectos femoris muscles both in WT and KO animals (not shown), but significantly decreased by 19% the gastrocnemius mass in both WT and KO animals, whereas 12 weeks of treatment with T3 only decreased the gastrocnemius mass in WT animals (19%), but not in KO animals (Figure 3C). Treatment with the supraphysiological dose of T3 (20xT3) promoted cardiac hypertrophy, evidenced by the increased dry mass of the heart (between 17 and 27%), in both WT and KO animals after 4 and 12 weeks of treatment (Figure 3D).

### Effect of T3 treatment on trabecular and cortical bone of the femur

**Figure 4:**
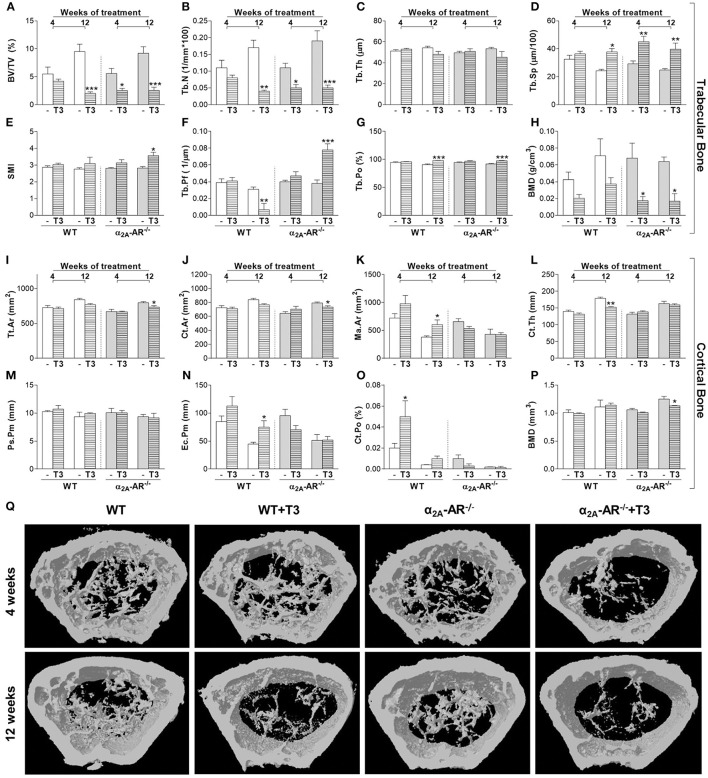
Effect of thyrotoxicosis on μCT parameters of trabecular and cortical bone of the femur in α_2A_-AR^−/−^ and WT mice. Animals were treated with a supraphysiological dose of T3 (7 μg/100 g body mass/day) or saline (-) for 4 or 12 weeks, by daily i.p. injections. Values are expressed as mean ± SEM (*n* = 7 per group). ^*^P < 0.05, ^**^p < 0.01, and ^***^p < 0.001 vs. the respective saline-treated mice, by Two-way ANOVA followed by Tukey's test. BV/TV, trabecular bone volume; Tb.N, trabecular number; Tb.Th, trabecular thickness; Tb.Sp, trabecular separation; SMI, structure model index; Tb.Pf, trabecular pattern function; Tb.Po, trabecular porosity; BMD, bone mineral density; Tt.Ar, total tissue area; Ct.Ar, cortical area; Ma.Ar, medullary area; Ct.Th, cortical thickness; Ps.Pm, periosteal perimeter; Ec.Pm, endocortical perimeter; and Ct.Po, cortical porosity. **(A–H)** Trabecular bone microarchitecture. **(I–P)** Cortical bone microarchitecture. **(Q)** μCT images of the distal methaphysis of the femur.

The μCT analysis of the femur showed that trabecular and cortical bone parameters were not different between WT and α2A-AR^−/−^ mice treated with saline (Figure 4). As expected, T3 treatment caused deleterious effects on both trabecular and cortical compartments of the femur, but trabecular bone of KO animals showed to be more sensitive to T3 effects than that of WT animals (Figures 4A–H). The 4-week treatment with T3 significantly decreased BV/TV (54%; Figure 4A), Tb.N (55%; Figure 4B), and trabecular BMD (74%; Figure 4H); and increased Tb.Sp (54%; Figure 4D) only in KO, but not in WT animals. The 12-week treatment with T3 significantly decreased BV/TV (Figure 4A) and Tb.N (Figure 4B), and increased Tb.Sp (Figure 4D) and Tb.Po (4G) in both WT and KO animals (78 and 72%, 76 and 73%, 55 and 59%, and 8 and 6%, respectively). However, this longer treatment increased SMI (27%; Figure 4E), which indicates an increase in rod-like trabeculae (vs. plate-like trabeculae); enhanced Tb.Pf (2.1-fold; Figure 4F), which indicates a lower trabecular connectivity; and decreased trabecular BMD (74%; Figure 4H) only in KO animals, and not in WT animals. Interestingly, some cortical bone parameters of the femur were more affected by thyrotoxicosis in WT animals, while others were more affected by T3 in α2A-AR^−/−^ animals (Figures 4I–Q). T3 treatment for 4 weeks practically had no effect on cortical bone of both WT and KO animals, except for Ct.Po, which was increased 2.5 times in WT mice, but not in KO animals. The longer T3 treatment (12 weeks) decreased Tt.Ar (8%), Ct.Ar (7%) and cortical BMD (10%) only in KO mice. On the other hand, the 12-week T3-treatment decreased Ct.Th (15%) and increased Ec.Pm (68%) and Ma.Ar (58%) only in the femur of WT animals, which suggests that TH-induced endocortical bone resorption depends on α2A-AR signaling.

### Effect of T3 treatment on trabecular and cortical bone of the vertebra (L5)

**Figure 5:**
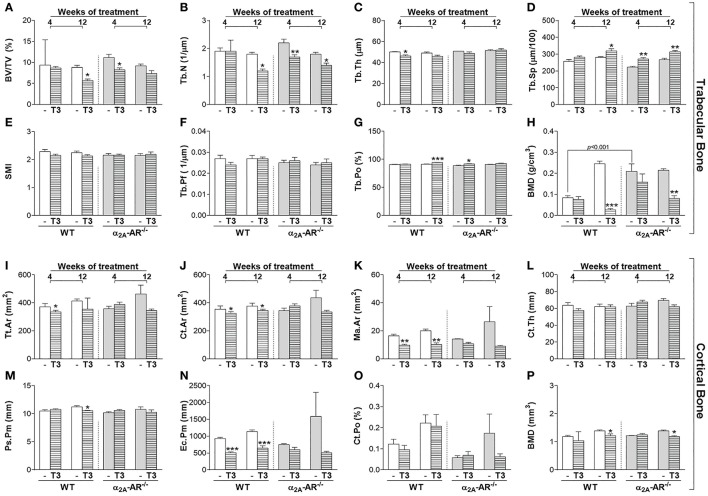
Effect of thyrotoxicosis on μCT parameters of trabecular and cortical bone of the vertebral body of L5 in α_2A_-AR^−/−^ and WT mice. Animals were treated with a supraphysiological dose of T3 (7 μg/100 g body mass/day) or saline (-) for 4 or 12 weeks, by daily i.p. injections. Values are expressed as mean ± SEM (*n* = 7 per group). ^*^P < 0.05, ^**^p < 0.01, and ^***^p < 0.001 vs. the respective saline-treated mice, by Two-way ANOVA followed by Tukey's test. BV/TV, trabecular bone volume; Tb.N, trabecular number; Tb.Th, trabecular thickness; Tb.Sp, trabecular separation; SMI, structure model index; Tb.Pf, trabecular pattern function; Tb.Po, trabecular porosity; BMD, bone mineral density; Tt.Ar, total tissue area; Ct.Ar, cortical area; Ma.Ar, medullary area; Ct.Th, cortical thickness; Ps.Pm, periosteal perimeter; Ec.Pm, endocortical perimeter; and Ct.Po, cortical porosity. **(A–H)** Trabecular bone microarchitecture. **(I–P)** Cortical bone microarchitecture.

Trabecular and cortical bone parameters of the vertebral body of L5 were not different between euthyroid WT and α2A-AR^−/−^ mice as well (Figure 5), except for trabecular BMD that was 2.5-fold higher in KO mice on the fourth week of this study, when animals were 58-day old, but this difference was no longer observed by the end of the study, when animals were 114 days old (Figure 5H). Trabecular bone of L5 also showed to be more sensitive to thyrotoxicosis in α2A-AR^−/−^ mice than in WT animals, but this difference was only observed after the shorter period of T3 treatment (4 weeks). T3 treatment for 4 weeks practically had no effect on the trabecular bone of L5 in WT mice, it only decreased Tb.Th (7%) in these animals (Figure 5C). In contrast, in α2A-AR^−/−^ mice, 4 weeks of T3 treatment decreased BV/TV (27%; Figure 5A) and Tb.N (22%; Figure 5B), and increased Tb.Sp (13%; Figure 5D) and Tb.Po (3%; Figure 5G). Twelve-weeks of T3 treatment was slightly more detrimental to WT mice. T3 increased Tb.Sp and decreased Tb.N and trabecular BMD in both WT and KO animals (13 and 17%, 33 and 23%, and 89 and 62%, respectively); but decreased BV/TV (35%) and increased Tb.Po (3%) only in WT animals (Figures 5A,G). In contrast to trabecular bone, cortical bone of L5 showed to be more sensitive to thyrotoxicosis in WT than in α2A-AR^−/−^ mice. T3 treatment decreased Tt.Ar (10% after 4 weeks), Ct.Ar (8% after 4 and 12 weeks), Ma.Ar (41 and 49% after 4 and 12 weeks, respectively), Ps.Pm (6% after 12 weeks) and Ec.Pm (45 and 43% after 4 and 12 weeks, respectively) only in WT animals (Figures 5I–K,N, respectively), but not in KO animals. BMD was decreased after 12 weeks of T3 treatment in both WT and KO animals in about 12% (Figure 5P). These data suggest that TH interacts with α2A-AR signaling to promote its detrimental effects on cortical bone of the vertebra.

### Effect of T3 treatment on biomechanical parameters of the femur

**Figure 6:**
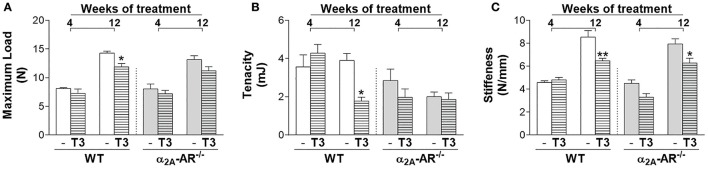
Effect of thyrotoxicosis on biomechanical parameters of the femur of α_2A_-AR^−/−^ and WT mice. Animals were treated with a supraphysiological dose of T3 (7 μg/100 g body mass/day) or saline (-) for 4 or 12 weeks, by daily i.p. injections. Values are expressed as mean ± SEM (*n* = 7 per group). ^*^P < 0.05 and ^**^p < 0.01 vs. the respective saline-treated mice, by Two-way ANOVA followed by Tukey's test.

Through the three-point bending test, we analyzed the effect of thyrotoxicosis on the femoral resistance to fracture. According to the μCT data, all the biomechanical parameters analyzed were not different between WT and α2A-AR^−/−^ animals. T3 treatment for 4 weeks had no effect on any parameter. On the other hand, when T3 treatment was extended to 12 weeks, it promoted a significant decrease in maximum load (17%) and tenacity (54%), only in WT animals and not in α2A-AR^−/−^ animals (Figures 6A,B); but significantly decreased stiffness (Figure 6C) in both WT and KO animals (24 and 21%, respectively). These findings suggested that α2A-AR signaling mediates the detrimental effects of TH on the femoral ability to resist fracture.

### Effect of T3 on mRNA expression of α2 and β adrenoceptors

**Figure 7:**
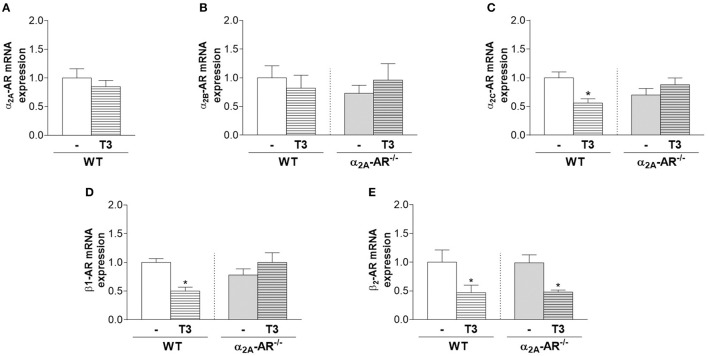
Effect of thyrotoxicosis on the relative mRNA expression of α_2A_-, α_2B_-, α_2C_-, β1- and β2-adrenoceptors in the femur. Animals were treated with a supraphysiological dose of T3 (7 μg/100 g body mass/day) or saline (-) for 12 weeks, by daily i.p. injections. mRNA expression in the whole femur was determined by real-time PCR analysis. Values are expressed as mean ± SEM (*n* = 7 per group). ^*^P < 0.05 vs. the respective saline-treated mice, by unpaired Student *t*-test or by Two-way ANOVA followed by Tukey's test.

T3 treatment had no effect on the mRNA expression of α2A-AR and α2B-AR (Figure 7A) in the whole femur of WT and/or KO mice (Figures 7A,B). On the other hand, T3 significantly decreased α2C-AR and β1-AR mRNA expression (44 and 50%, respectively) in the femur of WT mice, but not of α2A-AR^−/−^ mice (Figures 7C,D). On the other hand, β2-AR mRNA expression (Figure 7E) was decreased by T3 treatment in both WT and KO femurs (53 and 52%, respectively). Figure 7 also shows that the femoral mRNA expression of α2B-AR, α2C-AR, β1-AR and β2-AR is not different between WT and α2A-AR^−/−^ mice.

## Discussion

In the present study, we first characterized the skeletal phenotype of mice with single-gene inactivation of α2A-AR (α2A-AR^−/−^ mice), in an attempt to dissect the specific roles of α2-AR subtypes in bone physiology. Surprisingly, the μCT analysis of the trabecular and cortical bone parameters of the femur and vertebra (vertebral body of L5) showed no differences between α2A-AR^−/−^ mice and their WT controls in euthyroidism. These observations contrast with mice with global double-gene inactivation of α2A- and α2C-AR (α2A/C-AR^−/−^ mice), which exhibit a generalized HBM phenotype (28). These double KO mice present increased trabecular and cortical bone in the femur and vertebra, but mainly in the vertebra (vs. femur) and in the trabecular bone (vs. cortical bone) (Table 1), which initially suggested an osteopenic role of α2A- and/or α2C-AR signaling in the bone tissue. Accordingly, histomorphometric analysis showed that these animals present increased bone formation and decreased bone resorption, with higher BMD mainly in the vertebra (28). We later studied mice with global single-gene KO of α2C-AR (α2C-AR^−/−^ mice) (44). We found that α2C-AR^−/−^ mice present a HBM phenotype in the vertebra, with increased trabecular bone, but normal cortical bone (Table 1). The collective analysis of the vertebral phenotype of α2A/C-AR^−/−^ mice (increased trabecular and cortical bone) and α2C-AR^−/−^ mice (increased trabecular bone) indicated that α2C-AR signaling mediates osteopenic actions of the SNS on vertebral trabecular bone and suggested a negative action of α2A-AR signaling on vertebral cortical bone (28, 44). The present finding that euthyroid α2A-AR^−/−^ mice present a normal skeletal phenotype strengthens the role of α2C-AR signaling in mediating detrimental actions of the SNS on trabecular bone of the vertebra; on the other hand, it does not sustain, but does not exclude, a detrimental action of α2A-AR signaling on vertebral cortical bone.

**Table 1:** Trabecular and cortical bone microarchitecture and femoral resistance to fracture in α2-AR KO mouse models.

	**α2A/2C-AR^−/−^ (28)**	**α2C-AR^−/−^ (44)**	**α2A-AR^−/−^**
**VERTEBRA**			
Trabecular	↑BV/TV, ↑Tb.N ↓Tb.Pf and ↓SMI	↑BV/TV, ↑Tb.N, ↑Tb.Th ↓Tb.Sp	n.d.
Cortical	↑Ct.Th,↑BMD, ↑Ct.BV ↓Ma.Ar, ↓Ec.Pm	n.d.	n.d.
**FEMUR**			
Trabecular	↑BV/TV ↓Tb.Pf and ↓SMI	↓BV/TV, ↓Tb.N ↑Tb.Sp	n.d.
Cortical	↑BMD	↑Ma.Ar	n.d.
Fracture resistance	↑Maximum load	↓Maximum load	n.d.

*Bone microarchitecture was determined by μCT analysis and bone resistance to fracture by the three-point bending test. Arrows indicate if each parameter decreased (↓) or increased (↑) in KO mice vs. WT mice. n.d. indicates no difference vs. WT. Animals were 114 day-old when the tests were performed. μCT parameters: BV/TV, trabecular bone volume; Tb.N; trabecular number; Tb.Th, trabecular thickness; Tb.Sp, trabecular separation; Tb.Pf, trabecular pattern factor; SMI, structure model index; BMD, bone mineral density; Ct.Th, cortical thickness; Ct.BV, cortical bone volume; Ma.Ar, medullary area; Ec.Pm, endocortical perimeter. Biomechanical parameter: maximum load and resilience*.

In contrast to the HBM phenotype in the vertebra, α2C-AR^−/−^ mice showed a low bone mass (LBM) phenotype in the femur, with lower trabecular bone and nearly normal cortical bone (Table 1) (44). The lower trabecular content in the femur of α2C-AR^−/−^ mice raised two hypotheses: (i) α2C-AR signaling has anabolic actions on trabecular bone of the femur; and/or (ii) α2A-AR signaling has a predominant role in mediating osteopenic actions of the SNS on trabecular bone of the femur. Considering this latter hypothesis, one could expect increased trabecular bone in the femur of α2A-AR^−/−^ mice. However, the current study showed normal trabecular bone phenotype in the femur of euthyroid α2A-AR^−/−^ mice, which does not sustain a predominant catabolic role of α2A-AR signaling in euthyroid conditions, but supports anabolic actions of α2C-AR signaling on the trabecular compartment of the femur. On the other hand, negative actions of α2A-AR signaling on femoral trabecular bone cannot be excluded, as it will be discussed next.

**Figure 8:**
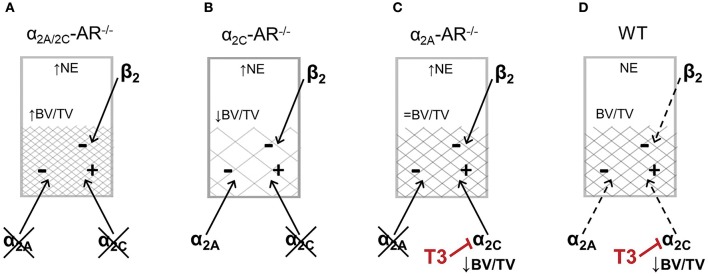
Schematic representation of a possible balance between α_2_-AR and β2-AR signaling to control femoral trabecular bone in the distal metaphysis of the femur in α_2A/2C_-AR^−/−^, α_2C_-AR^−/−^ and α_2A_-AR^−/−^ and WT mice. All KO mouse models present increased norepinephrine (NE) release and, therefore, increased sympathetic activation. **(A)** In α_2A/2C_-AR^−/−^ mice, the lack of α2A-AR predominantly results in anabolic actions that overcomes the catabolic effects of β2-AR activation and the lack of α_2C_-AR anabolic actions, resulting in increased trabecular bone volume (BV/TV). **(B)** In α2C-AR^−/−^ mice, the osteopenic actions of α2A-AR and β2-AR and the lack of α2C-AR-mediated anabolism results in lower BV/TV. **(C)** In α2A-AR^−/−^ mice, a balance between α2C-AR-mediated anabolism and β2-AR-mediated catabolism could result in normal BV/TV. **(D)** In WT mice, since NE release is normal, α2-AR and β2-AR are functioning at basal levels, and in equilibrium, to maintain bone mass. **(C,D)** T3 inhibits α2C-AR signaling, which suppresses the α2C-AR-mediated trabecular anabolism, leading to decreased BV/TV. ↑, ↓ and = vs. WT. This is a simplified scheme that does not includes other adrenoceptors that may mediate some of the effects observed in this study.

It is important to consider that all these mouse models present increased SNS outflow, since α2A-AR and α2C-AR are autoreceptors that inhibit the secretion of catecholamines (32, 33). The first one is the major presynaptic regulator of sympathetic NE release (32, 33), whereas α2C-AR is the main feedback receptor of adrenaline secretion from the chromaffin cells in the adrenal medulla (51), in addition to contribute to inhibition of NE release from sympathetic nerves (34). Therefore, it is important to consider that other adrenoceptors may be activated in these α2-AR KO mouse models. Evidence suggests that β2-AR is the main adrenoceptor to directly mediated SNS actions in the skeleton. Pharmacological blockade and activation of β2-AR signaling, respectively, increases and decreases bone mass (52–54), whereas global or osteoblast-specific genetic ablation of β2-AR in mice results in a HBM phenotype by 6 months of age (27, 55). Activation of β2-AR signaling inhibits osteoblast proliferation and activity and induces the expression of RANKL (receptor activator of nuclear factor kappa-B ligand), which binds to its receptor, RANK, in osteoclast precursor cells or in mature osteoclasts to activate osteoclastogenesis and to increase osteoclastic activity, respectively (27). On the other hand, β1-AR signaling seems to exert anabolic actions in the skeleton and, therefore, opposite effects in relation to β2-AR signaling. Nevertheless, euthyroid β1-AR^−/−^ mice show decreased trabecular bone in the vertebra but normal trabecular bone in the femur (56). There is also evidence that α1 adrenoceptors have a role in regulating osteoblast function (57, 58). Recently, α1B-AR signaling was shown to be required for bone formation and α1B-AR^−/−^ mice was shown to display reduced trabecular bone in the femur (59). Thus, the trabecular phenotypes in the femur of α2A/2C-AR^−/−^, α2C-AR^−/−^, and α2A-AR^−/−^ mice are expected to rely on a balance/unbalance among the actions of α2A-AR, α2C-AR and the other adrenoceptors, which adds great complexity to the actions of the SNS in the skeleton. Considering the well characterized actions of β2-AR signaling in the bone tissue, the increased trabecular bone in the femur of α2A/2C-AR^−/−^ mice suggests that lack of α2A-AR results in anabolic effects that could overcome the negative effects of β2-AR activation and the lack of α2C-AR-mediated anabolic actions (Figure 8A). On the other hand, in α2C-AR^−/−^ mice, the osteopenic actions of α2A-AR and β2-AR and the lack of α2C-AR-mediated anabolism result in lower trabecular bone content (Figure 8B). Finally, in α2A-AR^−/−^ mice (Figure 8C), a balance between α2C-AR-mediated anabolism and β2-AR-mediated catabolism could result in normal trabecular bone mass (as in WT controls). It is noteworthy that in the present study, the mRNA expression of α2B-, α2C-, β1 and β2-adrenoceptors was not different between WT and KO animals, but, as discussed above, it is expected that the activation of these receptors differs between WT and KO mice. Further studies are necessary to investigate the actions and interactions among all these adrenoceptors to control bone remodeling.

Regarding the femoral cortical bone, α2A-AR^−/−^ mice present no alterations; α2A/2C-AR^−/−^ mice present an increase only in volumetric BMD (28), suggesting negative actions of α2-AR signaling in this parameter; whereas α2C-AR^−/−^ mice present an increase in Ma.AR (44), which is suggestive of increased endosteal resorption. This finding suggests anabolic actions of α2C-AR signaling also in the cortical bone of the femur. Accordingly, α2A-AR^−/−^ mice show normal (same as WT mice) resistance to fracture whereas α2C-AR^−/−^ mice display reduced resistance to fracture (Table 1), determined by the three-point bending test (decreased maximum load and resilience). It is noteworthy that this test measures mostly the resistance of the femoral diaphysis to fracture, which is a skeletal site mainly composed by cortical bone. These findings, therefore, support a positive role of α2C-AR signaling in the cortical bone and in its ability to resist fracture.

To add insights to our understanding about the interaction of TH with the SNS to control bone physiology, and to clarify the specific roles of α2-AR subtypes in this process, we evaluated the bone responses of α2A-AR^−/−^ mice to 4 and 12 weeks of daily treatment with 20xT3. This daily treatment increased serum levels of T3 and decreased serum levels of T4 in both WT and KO mice, which reflects the suppression of the HPT axis by TH excess (50), and confirms a thyrotoxic state in these animals. To further confirm a thyrotoxic state, we investigated the effect of T3 treatment on body composition. TH excess for 4 and 12 weeks promoted the characteristic effects of thyrotoxicosis in WT mice, including some decrease in BW, fat mass (axillar and retroperitoneal fat pads) and muscle mass (gastrocnemius mass). Besides, this T3 treatment increased heart mass, which reflects cardiac hypertrophy, a known consequence of toxic levels of TH (60, 61). Responses of α2A-AR^−/−^ mice to thyrotoxicosis were different from those of WT mice in several parameters, including bone parameters, as it will be discussed below. KO animals showed an 8–13% lower BW than WT mice from 58 days of age until the end of the study (8 weeks later), which is probably explained by increased lipolysis. There is evidence that α2-AR signaling has antilipolytic actions (62, 63). Thus, the increased NE release and the impaired α2-AR-dependent antilipolysis are likely to facilitate the lipolytic action of β-AR (64). Accordingly, α2A-AR^−/−^ animals presented lower retroperitoneal fat pad than WT mice. Surprisingly, TH failed to promote reductions in the retroperitoneal and axillar fat pads in KO animals, suggesting a complex role of α2A-AR in lipolysis, which remains to be investigated. Heart mass was increased in 58-day-old saline-treated α2A-AR^−/−^ mice (vs. WT mice), which reflects cardiac hypertrophy because of the enhanced sympathetic activity (34). Nevertheless, TH promoted cardiac hypertrophy in α2A-AR^−/−^ mice as much as in WT mice, further confirming a thyrotoxic state in both mouse lineages.

Regardless of the normal bone phenotype in euthyroid α2A-AR^−/−^ mice, in general, trabecular bone showed to be more sensitive to thyrotoxicosis, whereas cortical bone showed to be resistant to the osteopenic effects of thyrotoxicosis in these KO animals, when compared with their WT controls. In order to gain insights into the role of each α2-AR subtypes on the TH-SNS interaction to control bone physiology, we compared these new findings with the already published data of α2A/C-AR^−/−^ and α2C-AR^−/−^ mice (28, 37, 44). The higher sensitivity of trabecular bone to TH in α2A-AR^−/−^ mice contrasts the lower sensitivity of trabecular bone to TH in α2A/C-AR^−/−^ and α2C-AR^−/−^ mice (37, 44), which suggests that α2C-AR signaling has a key role in the mechanism by which TH excess promotes its detrimental effects on trabecular bone. On the other hand, cortical bone of all these KO models showed some degree of resistance to thyrotoxicosis (37, 44), suggesting that α2A-AR and α2C-AR signaling contributes to TH actions on cortical bone.

The current study showed that TH excess for 4 weeks decreased BV/TV, Tb.N and increased Tb.Sp and Tb.Po only in the vertebra of α2A-AR^−/−^ mice, whereas WT mice showed only a reduction in Tb.Th. However, after 12 weeks of TH treatment, vertebral trabecular bone of WT mice showed to be slightly more sensitive to the detrimental effects of thyrotoxicosis than α2A-AR^−/−^ mice, since 20xT3 decreased BV/TV and increased Tb.Po only in WT animals. Conciliating these new findings with the observation that vertebral trabecular bone of α2C-AR^−/−^ mice shows resistance to thyrotoxicosis after 4 and 12 weeks of T3 treatment (44), we could suppose that α2C-AR signaling is the main α2-AR subtype to mediate TH actions on trabecular bone of the vertebra until 4 weeks of thyrotoxicosis, but that, in longer situations of thyrotoxicosis (12 weeks), α2A-AR signaling could also contribute to the detrimental effects of TH excess. Interestingly, cortical bone of the vertebra showed to be more sensitive to thyrotoxicosis in WT than in α2A-AR^−/−^ mice. Thyrotoxicosis negatively affected Ct.Ar, Ma.Ar, Ps.Pm, and Ec.Pm in WT mice and not in α2A-AR^−/−^ mice. Similar effects of toxic levels of T3 were also observed in α2A/2C-AR^−/−^ mice (37), which were resistant to TH-induced decreases in Ct.Th, T.Ar, Ct.Ar, Ct.BV, and Ma.Ar, and to TH-induced increases in Ct.Po. and in α2C-AR^−/−^ mice (44), which were resistant to TH-induced decreases in T.Ar and increases in Ec.Pm.^,^ Altogether, these findings suggest that both α2A-AR and α2C-AR signaling pathways mediate TH actions on the cortical compartment of the vertebra.

The mechanisms by which TH interacts with the SNS to control bone morphophysiology in the femur seems to be different than that in the vertebra. As discussed before, α2A/2C-AR^−/−^ mice present increased trabecular (mainly) and cortical bone in the femur (37); α2C-AR^−/−^ mice present lower trabecular bone and nearly normal femoral cortical bone (44); whereas α2A-AR^−/−^ mice present normal trabecular and cortical bone in the femur (Table 1). Considering these findings, anabolic roles of α2C-AR signaling in the trabecular compartment of the femur have emerged. The study of α2A/2C-AR^−/−^ and α2C-AR^−/−^ mice showed that trabecular and cortical compartments of the femur are resistant to detrimental effects of thyrtotoxicosis (37, 44). In contrast, the present study shows that thyrotoxicosis was clearly more deleterious to the trabecular bone of the femur in α2A-AR^−/−^ mice than in WT mice, whereas cortical bone of the femur was less sensitive to thyrotoxicosis in α2A-AR^−/−^ mice. Altogether, these studies suggest that α2C-AR signaling, but not α2A-AR, is necessary for TH to promote its detrimental effects in the trabecular bone of the femur, whereas both α2A-AR and α2C-AR mediate TH effects in the femoral cortical bone.

The three-point bending test showed that thyrotoxicosis decreased femoral resistance to fracture (lower maximum load and tenacity) in WT mice, but not in α2A-AR^−/−^ animals. This finding is consistent with the more deleterious effects of thyrotoxicosis in the cortical bone of WT mice. Similar results were observed in α2A/2C-AR^−/−^ and α2C-AR^−/−^, which also showed resistance to the detrimental effects of TH on bone ability to resist fracture. These findings, therefore, support a role of both α2A-AR and α2C-AR signaling pathways in mediating detrimental actions of TH on cortical bone of the femur.

The fact that α2C-AR signaling arises as having anabolic actions on trabecular and cortical femoral bone while seems to be necessary for TH to promote its deleterious effects in the femur is intriguing. We, therefore, suggest that one mechanism by which TH promote its deleterious effects in the femur is decreasing α2C-AR signaling (Figures 8C,D), which would decrease bone anabolism and favor catabolism. Accordingly, we found that T3 decreases α2C-AR mRNA expression in the femur of WT mice. This effect, however, was not observed in α2A-AR^−/−^ mice. It is important to consider that TH actions on the expression of adrenoceptors are normally modest or nonexistent, and usually cannot explain the T3-SNS interactions (38). Evidence shows that TH usually modulates more distal cellular effectors in the adrenoceptors signaling pathways (38). Further studies will be necessary to confirm if T3 really suppresses α2C-AR signaling in bone cells and in which level it occurs. We also found that T3 had no effect on α2A- and α2B-AR mRNA expression. On the other hand, T3 decreased femoral mRNA expression of β1-AR in WT but not in α2A-AR^−/−^ mice, whereas decreased β2-AR mRNA expression in both WT and α2A-AR^−/−^ mice. The relevance of these modulations cannot be extrapolated at this moment, but the effects of T3 on the expression of these adrenoceptors suggest that the modulation of adrenoceptor signaling may be a point of interaction between TH and the SNS to regulate bone mass.

It is important to consider that besides their function as autoreceptors, α2-AR subtypes are also expressed in non-adrenergic neurons, where they can operate as heteroreceptors to regulate the release of several neurotransmitters in the central and peripheral nervous system, including serotonin, GABA and dopamine, among other neurotransmitters (65). In addition to neuronal locations, α2-AR subtypes are also present in several non-neuronal cells/tissues (vascular vessels, pancreatic islets, etc.), which actions have emerged as relevant to the body (65). The widespread expression of α-AR subtypes in the central and peripheral nervous system, and in non-neuronal cells adds further complexity to the understanding of the roles of α2-AR signaling in several physiological processes, including bone metabolism. An important limitation of the present study is that it is based on mouse models of global gene inactivation of α2-AR subtypes. Thus, the interference of factors from the central and peripheral nervous system, as well as interferences from systemic and local factors may have occurred in the bone effects of TH observed in the present study. In addition, these global mouse models do not allow the discrimination of the contribution of central and local TH-SNS interactions. Nevertheless, we have previously shown that clonidine (CLO), an α2-AR agonist, increases osteoclastogenesis, *in vitro*, in mouse marrow cells derived from WT animals, but not from α2A/C-AR^−/−^ mice. In contrast, phentolamine, a nonspecific α-AR antagonist, decreases osteoclast formation in cultures of marrow cells derived from WT but not in the same cells derived from α2A/C-AR^−/−^ animals (28). In addition, further *in vitro* studies showed that CLO or T3 alone decreased proliferation of calvaria-derived osteoblasts isolated from WT mice, which was further decreased when cells were treated with CLO combined with T3 (CLO+T3). These effects, however, were completely blocked or reversed in α2A/2C-AR^−/−^ mice-derived cells (37). These *in vitro* studies and the expression of all α2-AR isotypes in the main skeletal cells (28) support direct actions of the SNS on the skeleton and show that a TH-SNS interaction is likely to occur locally in the skeleton, via α2A-AR and/or α2C-AR signaling. Thus, these studies suggest that one mechanism by which TH regulates bone physiology directly in the skeleton involves a local interaction with α2-AR signaling.

In summary, euthyroid α2A-AR^−/−^ mice present normal trabecular and cortical bone in the femur and vertebra, in addition to present normal resistance to fracture. The combined analysis of the bone phenotypes of α2A/C-AR^−/−^, αC-AR^−/−^, and α2A-AR^−/−^ mice suggests that: (i) α2-AR signaling mediates SNS-actions mainly in trabecular bone; (ii) α2C-AR signaling predominantly mediates osteopenic actions of the SNS on trabecular bone of the vertebra; (iii) α2C-AR signaling has anabolic actions mainly on trabecular bone but also on cortical bone of the femur. Regardless of the normal bone phenotype in euthryoidism, α2A-AR^−/−^ mice responds differently than WT mice to thyrotoxicosis. The higher sensitivity of trabecular bone to TH in α2A-AR^−/−^ mice and the lower sensitivity of trabecular bone to TH in α2A/C-AR^−/−^ and α2C-AR^−/−^ mice (37, 44) points out α2C-AR signaling as a key factor to mediate the detrimental actions of TH on trabecular bone, particularly in the femur. On the other hand, the lower sensitivity of cortical bone to thyrotoxicosis in α2A/C-AR^−/−^, α2C-AR^−/−^, and α2A-AR^−/−^ mice (37, 44) suggests that both α2A-AR and α2C-AR signaling pathways contribute to detrimental actions of TH on cortical bone of both femur and vertebra. Altogether, these novel findings further sustain a TH-SNS interaction, involving α2-AR signaling, to regulate bone remodeling, and, therefore, bone mass, bone integrity and, ultimately, bone resistance to fracture.

## Author contributions

GM: Conceived and performed experiments, carried out data collection and analysis, and wrote the manuscript; MT: performed experiments, carried out data collection and analysis, and reviewed the manuscript; MS, BN-P, and MM-R: performed experiments, carried out data collection, and reviewed the manuscript; PB: Conceived the study and experiments, carried out data analysis and reviewed the manuscript; CG: Conceived the study and experiments, carried out data analysis and wrote the manuscript.

### Conflict of interest statement

The authors declare that the research was conducted in the absence of any commercial or financial relationships that could be construed as a potential conflict of interest.
